# Trastuzumab deruxtecan in HER2-positive advanced gastric cancer: exploratory biomarker analysis of the randomized, phase 2 DESTINY-Gastric01 trial

**DOI:** 10.1038/s41591-024-02992-x

**Published:** 2024-05-14

**Authors:** Kohei Shitara, Yung-Jue Bang, Satoru Iwasa, Naotoshi Sugimoto, Min-Hee Ryu, Daisuke Sakai, Hyun Cheol Chung, Hisato Kawakami, Hiroshi Yabusaki, Yasuhiro Sakamoto, Tomohiro Nishina, Koichiro Inaki, Yusuke Kuwahara, Naoya Wada, Fumitaka Suto, Takeo Arita, Masahiro Sugihara, Zenta Tsuchihashi, Kaku Saito, Akihito Kojima, Kensei Yamaguchi

**Affiliations:** 1 National Cancer Center Hospital East, Kashiwa, Japan; 2 Department of Immunology, Nagoya University Graduate School of Medicine, Nagoya, Japan; 3 Seoul National University College of Medicine, Seoul, Republic of Korea; 4 National Cancer Center Hospital, Tokyo, Japan; 5 Osaka International Cancer Institute, Osaka, Japan; 6 Asan Medical Center, University of Ulsan College of Medicine, Seoul, Republic of Korea; 7 Yonsei Cancer Center, Yonsei University College of Medicine, Seoul, Republic of Korea; 8 Kindai University Hospital, Osaka, Japan; 9 Niigata Cancer Center Hospital, Niigata, Japan; 10 Osaki Citizen Hospital, Osaki, Japan; 11 National Hospital Organization Shikoku Cancer Center, Ehime, Japan; 12 Daiichi Sankyo Co. Ltd, Tokyo, Japan; 13 Daiichi Sankyo Inc., Basking Ridge, NJ USA; 14 Daiichi Sankyo RD Novare Co. Ltd, Tokyo, Japan; 15 Cancer Institute Hospital of Japanese Foundation for Cancer Research, Tokyo, Japan

**Keywords:** Predictive markers, Gastric cancer

## Abstract

Trastuzumab deruxtecan (T-DXd) showed statistically significant clinical improvement in patients with human epidermal growth factor receptor 2-positive (HER2^+^) gastric cancer in the DESTINY-Gastric01 trial. Exploratory results from DESTINY-Gastric01 suggested a potential benefit in patients with HER2-low gastric cancer. Spatial and temporal heterogeneity in HER2 expression or gene alteration, an inherent characteristic of gastric cancer tumors, presents a challenge in identifying patients who may respond to T-DXd. Specific biomarkers related to therapeutic response have not been explored extensively. Exploratory analyses were conducted to assess baseline HER2-associated biomarkers in circulating tumor DNA and tissue samples, and to investigate mechanisms of resistance to T-DXd. Baseline HER2-associated biomarkers were correlated with objective response rate (ORR) in the primary cohort of patients with HER2^+^ gastric cancer. The primary cohort had 64% concordance between HER2 positivity and *HER2* (*ERBB2*) plasma gene amplification. Other key driver gene amplifications, specifically *MET*, *EGFR* and *FGFR2*, in circulating tumor DNA were associated with numerically lower ORR. Among 12 patients with *HER2* gain-of-function mutations, ORR was 58.3% (7 of 12). ORR was consistent regardless of timing of immunohistochemistry sample collection. Further investigations are required in larger studies.

## Abstract (web summary)

Exploratory analyses of the DESTINY-Gastric01 trial show that baseline HER2-associated biomarkers in circulating tumor DNA or tissue samples were associated with therapeutic response in patients with HER2-positive tumors, and these analyses identify potential drivers of resistance.

## Main

Trastuzumab deruxtecan is an antibody–drug conjugate (ADC) comprising an antihuman HER2 antibody, a tetrapeptide-based cleavable linker and a topoisomerase I inhibitor payload^1^. In clinical trials T-DXd has shown activity in a variety of cancers, including gastric and gastroesophageal junction (GEJ) cancer^2^. DESTINY-Gastric01 was a pivotal randomized study of T-DXd versus third- or later-line chemotherapy in patients with HER2^+^ gastric or GEJ tumors.

In the DESTINY-Gastric01 primary cohort of patients with HER2^+^ gastric cancer centrally confirmed as immunohistochemistry (IHC) score 3^+^ or IHC 2^+^/in situ hybridization (ISH)^+^ using the most recent archival or fresh biopsy tumor tissues, T-DXd significantly improved ORR compared with physician choice of chemotherapy (TPC; ORR 51% (95% confidence interval (CI)^3^ 42–61%) versus 14% (95% CI 6–26%)) and led to significantly improved overall survival (OS; median OS 12.5 months (95% CI 9.6–14.3 months) versus 8.4 months (95% CI 6.9–10.7 months))^4^. These findings supported the regulatory approval of T-DXd by (1) the US Food and Drug Administration, (2) the Ministry of Health, Labor, and Welfare of Japan and (3) the European Medicines Agency for previously treated, locally advanced or metastatic HER2^+^ gastric and GEJ cancer^4^. T-DXd activity was also suggested in the HER2-low (IHC 2^+^/ISH^−^ or IHC 1^+^) exploratory cohorts of DESTINY-Gastric01, with a confirmed ORR (ORR lasting ≥4 weeks) of 26.3% (95% CI 9.1–51.2%) for IHC 2^+^/ISH^−^ and 9.5% (95% CI 1.2–30.4%) for IHC 1^+^ (ref. ^5^). The activity of T-DXd was further confirmed in the phase 2 DESTINY-Gastric02 study in patients from the United States and Europe; this study enrolled patients with HER2^+^ status, measured using fresh biopsy samples obtained following trastuzumab treatment^6^. T-DXd is now recommended as a second- or third-line treatment following trastuzumab treatment^3,7^. Furthermore, T-DXd versus second-line paclitaxel plus ramucirumab is being investigated in the ongoing phase 3 DESTINY-Gastric04 study (NCT04704934).

Spatial heterogeneity (heterogeneous HER2 expression and concomitant alterations) and temporal heterogeneity (loss of HER2 expression and acquired alterations) may make it challenging to identify patients with gastric or GEJ cancer who have the potential to respond to T-DXd treatment. Intratumoral heterogeneity is a more common phenomenon in HER2^+^ gastric cancer compared with HER2^+^ breast cancer^8,9^. Moreover, decreased HER2 expression following treatment with trastuzumab or other HER2-targeted agents has been observed in 16–32% of patients^10–12^. Circulating tumor DNA (ctDNA) analysis is a noninvasive and convenient method widely used in clinics to detect gene alterations in patients with cancer^13^. However, the relationship between gene alterations in ctDNA and the efficacy of ADCs that target specific oncoproteins has not been thoroughly evaluated. The objective of the present analyses was to identify patients who may benefit from treatment with T-DXd based on relevant biomarkers in tumor or ctDNA.

## Results

### Relationship between baseline HER2 levels and response

**Fig. 1:**
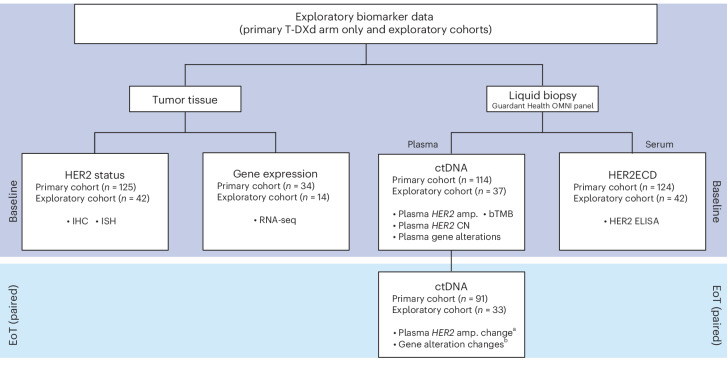
Sample collection scheme. Exploratory biomarker data were collected from primary and exploratory cohorts at baseline and EoT. Tumor tissue was selected for HER2 status and RNA-seq, and liquid biopsy was used to examine plasma and serum biomarkers. Plasma analysis examined ctDNA and serum biomarkers included HER2ECD. There were 91 patients in the HER2 IHC 3^+^ cohort and 28 in the HER2 IHC 2^+^/ISH^+^ cohort. ^a^Changes in plasma *HER2* amplification were categorized as either baseline (alterations detected at baseline) or acquired (alterations detected only in EoT samples). ^b^Gene alterations concomitant with T-DXd resistance. Amp., amplification; CGEP, comprehensive gene expression profile.

All patients from DESTINY-Gastric01 (125 from the primary cohort, 42 from the exploratory cohorts) had samples available for measurement of baseline HER2 status, and most patients (114 in the primary cohort, 37 in the exploratory cohorts) had samples available for ctDNA analyses. However, only some tissue biopsy samples taken just before study treatment were available for analyses (34 of 125 for the primary cohort, 14 of 42 for the exploratory cohorts) (Fig. 1).

**Fig. 2:**
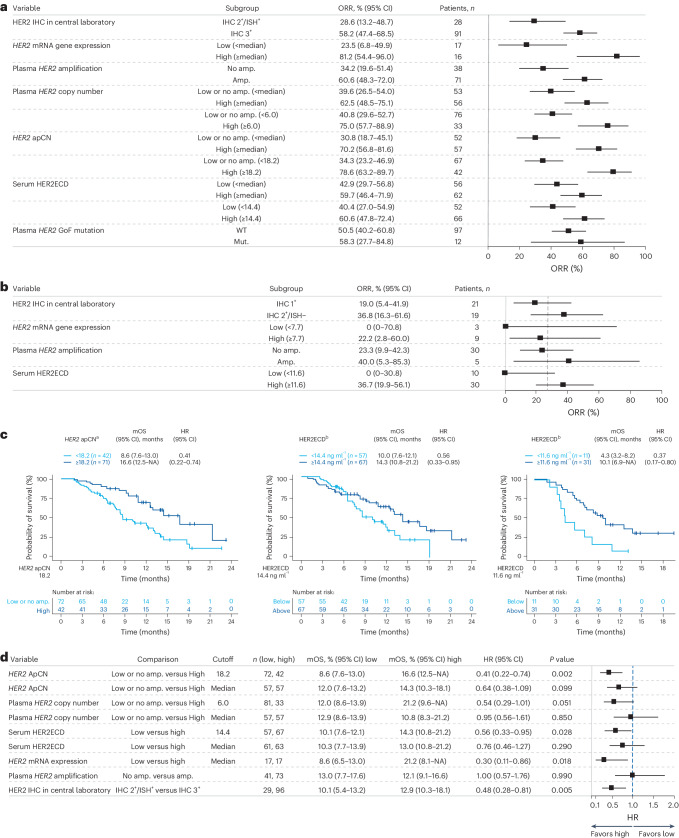
Baseline HER2 expression in primary and exploratory cohorts. **a**, Univariate analysis of baseline HER2 biomarker status and ORR in the primary cohort T-DXd arm. **b**, ORR based on biomarker status in the exploratory cohorts (HER2-low). **c**, OS by adjusted plasma *HER2* copy number, HER2ECD in the primary cohort and HER2ECD in the exploratory cohorts. **d**, Forest plot of OS. **a**, *HER2* mRNA gene expression median 9.72; serum HER2ECD median 9.72. **b**, Dashed vertical line represents overall ORR. **a**,**b**, Error bars represent 95% CI. **d**, HR < 1 favors the biomarker-selected group. Error bars represent 95% CI. ^a^For OS by *HER2* ApCN, an exploratory cutoff (apCN 18.2) value was determined that minimized the *P* value estimated by log-rank test. Patients with values below 18.2 included those with no amplification. ^b^For OS according to HER2ECD, an exploratory cutoff value (14.4 ng ml^−1^ in the primary cohort, 11.6 ng ml^−1^ in the exploratory cohorts) was determined that minimized the *P* value estimated by log-rank test. Mut, mutant.

Univariate analysis of baseline HER2 status in the primary cohort (IHC 3^+^ or IHC 2^+^/ISH^+^) biomarker analysis, as measured by IHC, RNA expression, amplification copy number (CN) in ctDNA and serum HER2 extracellular domain (HER2ECD), demonstrated a consistent trend of increased ORR in patients with higher HER2 status (Fig. 2a). In this analysis, patients with centrally assessed tumor HER2 IHC 3^+^ status in the primary cohort (*n* = 91) had higher ORR than those with HER2 IHC 2^+^/ISH^+^ status (*n* = 28; ORR (95% CI) 58.2% (47.4–68.5%) versus 28.6% (13.2–48.7%), respectively) (Fig. 2a). Patients with high *HER2* (*ERBB2*) messenger RNA (mRNA) gene expression (defined as equal to or greater than the median value of 9.72) (*n* = 16) had higher ORR compared with those with low *HER2* mRNA gene expression (defined as below the median value of 9.72) (*n* = 17): (ORR (95% CI) 81.2% (54.4–96.0%) versus 23.5% (6.8–49.9%)) as assessed by RNA sequencing (RNA-seq). The subset of patients with plasma *HER2* amplification in ctDNA (*n* = 71) had higher ORR compared with those without amplification (*n* = 38) (ORR (95% CI) 60.6% (48.3–72.0%) versus 34.2% (19.6–51.4%)) (Fig. 2a). Patients with high adjusted plasma copy number (apCN) for *HER2* (defined by an exploratory cutoff value of ≥18.2 that minimized the *P* value estimated by log-rank test for OS; *n* = 42) had higher ORR compared with patients with low *HER2* apCN (defined as below the exploratory cutoff value of 18.2; *n* = 67) (ORR (95% CI) 78.6% (63.2–89.7%) versus 34.3% (23.2–46.9%)). Patients with high serum HER2ECD concentration (defined as equal to or greater than the median value of 9.72; *n* = 62) had higher ORR compared with those with low serum HER2ECD (defined as below the median value of 9.72; *n* = 56); ORR (95% CI) 59.7% (46.4–71.9%) versus 42.9% (29.7–56.8%) (Fig. 2a). *HER2* gain-of-function (GoF) variants were detected in 11.0% (12 of 109) of response-evaluable patients. Most patients with *HER2* GoF mutations also had *HER2* amplifications (one of 13 for aneuploidy and 11 of 13 for focal amplification), and all had HER2 IHC 3^+^ or IHC 2^+^/ISH^+^ status (Extended Data Table 1). The ORR for patients with HER2 IHC 3^+^ status and *HER2* GoF mutation was 87.5% (seven of eight) versus 58.2% (53 of 91) for patients with HER2 IHC 3^+^ status in the overall population; however, sample sizes were too small for meaningful comparison.

In the exploratory HER2-low cohorts, the ORR of patients with baseline HER2ECD level greater than or equal to the exploratory cutoff value of 11.6 ng ml^−1^ (*n* = 30), which minimized the *P* value estimated by log-rank test for OS, was higher at 36.7% (95% CI 19.9–56.1%) compared with 0% (95% CI 0–30.8%) for patients with baseline HER2ECD <11.6 ng ml^−1^ (*n* = 10) (Fig. 2b).

In the primary cohort, median OS (mOS) was longer in patients with high *HER2* apCN (defined as apCN equal to or greater than the exploratory cutoff value of 18.2 that minimized the *P* value estimated by log-rank test for OS) compared with patients with low *HER2* apCN (defined as apCN <18.2) (mOS (95% CI) 16.6 months (12.5 months–not available (NA)) versus 8.6 months (7.6–13.0 months)) (Fig. 2c). Longer mOS was also observed in patients in the primary cohort when high *HER2* apCN (≥18.2) was defined as being higher than the median value (Fig. 2d). mOS in the primary cohort was longer in patients with high HER2ECD levels (defined as equal to or greater than the exploratory cutoff value of 14.4 ng ml^−1^, which minimized the *P* value estimated by log-rank test; *n* = 67) compared with patients with low HER2ECD levels (defined as <14.4 ng ml^−1^; *n* = 57) (mOS (95% CI) 14.3 months (10.8–21.2 months) versus 10.0 months (7.6–12.1 months)) (Fig. 2c). In the exploratory cohorts, longer mOS was observed in patients with high HER2ECD versus those with low HER2ECD (mOS (95% CI) 10.1 months (6.9 months–NA) in patients with HER2ECD ≥11.6 ng ml^−1^, the exploratory cutoff that minimized the *P* value estimated by log-rank test, versus 4.3 months (3.2–8.2 months) in patients with HER2ECD <11.6 ng ml^−1^) (Fig. 2c).

### HER2 biomarkers in ctDNA and HER2 expression in tissue

**Extended Data Fig. 1:**
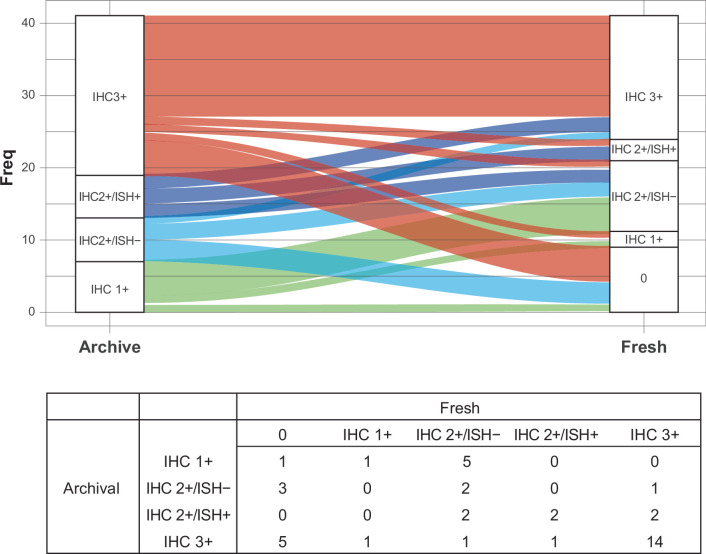
HER2 status between matched archive and fresh biopsy samples. Freq, frequency; HER2, human epidermal growth factor receptor 2; IHC, immunohistochemistry; ISH, in situ hybridization; T-DXd, trastuzumab deruxtecan. All samples (fresh and archival) were taken before T-DXd treatment. There were 41 samples included in this analysis. Two of the 41 paired samples were collected from non-matched sites. One archive sample was collected from ‘other’ as the site; this sample had a HER2 IHC status change from HER2 1+ to HER2 2 + /ISH−. Another archive sample was collected from liver as the site; this sample did not have a HER2 IHC status change (HER2 IHC 3+ to HER2 IHC 3+).

High concordance was observed between plasma *HER2* amplification in ctDNA and HER2 tissue expression (Extended Data Table 2). The positive predictive agreement (PPA) between tumor HER2 status (positive, IHC 3^+^ or IHC 2^+^/ISH^+^; low, IHC 2^+^/ISH^−^ or IHC 1^+^) and plasma amplification in ctDNA was 64% and the negative predictive agreement (NPA) was 86% (Extended Data Table 2). For the fresh biopsy tissue samples used for trial enrollment, positive predictive agreement was 69% (nine of 13) and NPA was 100% (five of five). Comparative HER2 status between archival and fresh-tissue biopsy samples taken for exploratory biomarker analysis is shown in Extended Data Fig. 1. In this analysis, 24.4% of patients reported a HER2 status change from archival to fresh tissue; the HER2 status of nine of 28 patients (32.1%) was downgraded from HER2^+^ to HER2^−^ and that of one of 13 patients (7.7%) was upgraded from HER2^−^ to HER2^+^.

**Fig. 3:**
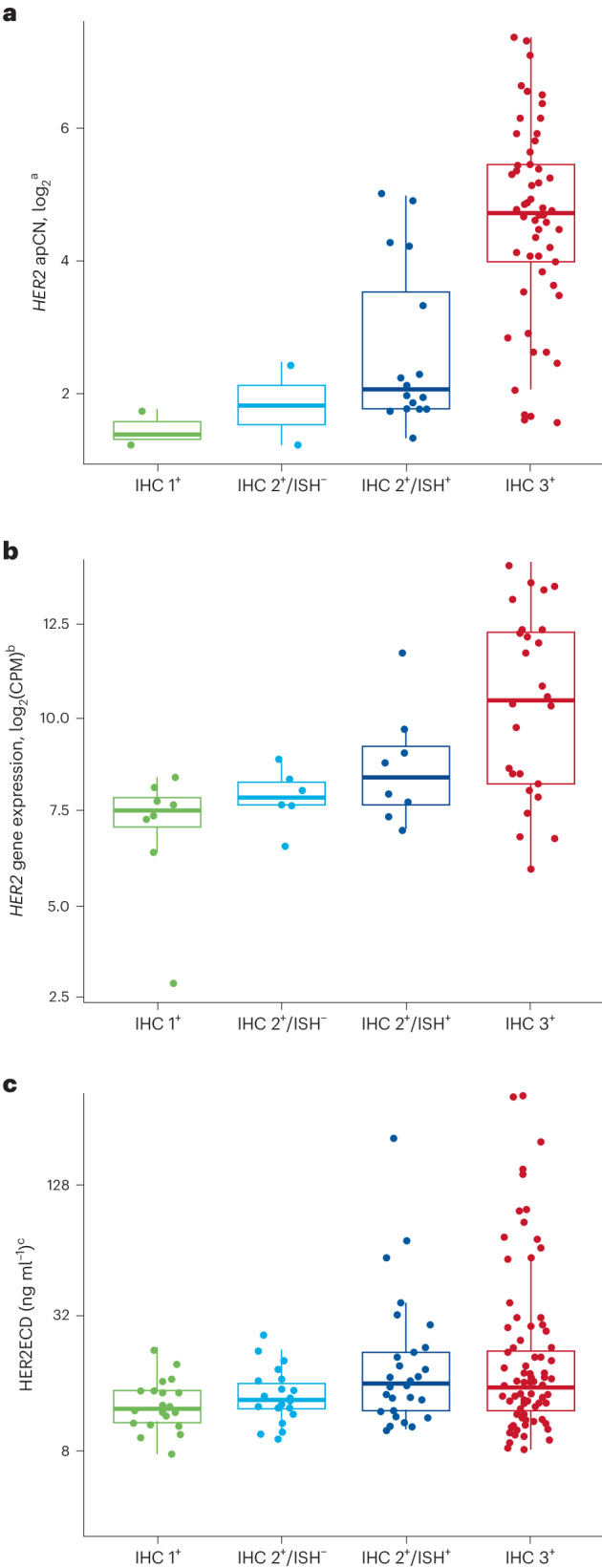
HER2 status in ctDNA plasma and tumor samples. **a**–**c**, Samples were assessed by *HER2* apCN (**a**), *HER2* (**b**) and serum HER2ECD (**c**). Minimum and maximum are represented by the whiskers, the box represents the first–third quarters, the center represents the median and dots represent individual samples. ^a^Only HER2 plasma-detected samples are shown; all cohorts are included except for two patients from the exploratory cohorts whose HER2 test results were missing. ^b^Baseline only (48 samples); all cohorts are included. ^c^Baseline only (166 samples); all cohorts are included except for two patients from the exploratory cohorts whose HER2 test results were missing. CPM, counts per million.

ctDNA plasma *HER2* apCN was associated with HER2 status assessed in the tumor by IHC/ISH (Fig. 3a). Higher tumor HER2 levels (HER2^+^ assessed with IHC/ISH) were associated with higher *HER2* apCN. *HER2* mRNA expression level and HER2ECD were also associated with tumor HER2 status (HER2^+^ assessed with IHC/ISH) (Fig. 3b,c).

### Clinical activity based on time of tumor sample collection

**Fig. 4:**
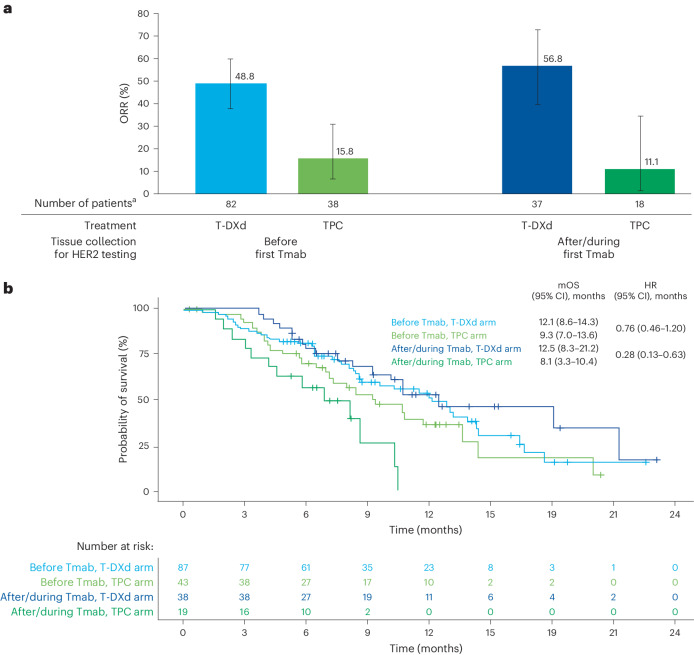
Clinical outcomes in the primary cohort according to timing of tissue collection with respect to first Tmab treatment. **a**, ORR in the study according to tissue collected before or after/during first Tmab treatment. **b**, OS analyzed over the course of the study according to tissue collected before or after/during first Tmab treatment. **a**, Error bars represent 95% CI; the center of error bars indicates ORR. ^a^Includes data for the response-evaluable set (all randomized patients who received at least one dose of study drug and had measurable tumors based on independent central review at baseline).

T-DXd activity was demonstrated in the primary cohort, regardless of the timing of tissue collection for assessment of HER2 status with respect to the first treatment with trastuzumab monoclonal (Tmab)—that is, before the first Tmab treatment versus after/during the first Tmab treatment (Fig. 4a). The ORR in the T-DXd arm was 48.8% (40 of 82) in patients who had IHC sample collection taken at any time before their first Tmab treatment and 56.8% (21 of 37) in patients who had IHC sample collection taken after/during their first Tmab treatment. In patients with IHC samples collected before the first Tmab treatment, mOS was 12.1 months (95% CI 8.6–14.3 months) in the T-DXd arm and 9.3 months (95% CI 7.0–13.6 months) in the TPC arm (hazard ratio (HR) 0.76, 95% CI 0.46–1.20) (Fig. 4b). In patients with IHC samples collected during/after the first Tmab treatment, mOS was 12.5 months (95% CI 8.3–21.2 months) in the T-DXd arm and 8.1 months (95% CI 3.3–10.4 months) in the TPC arm (HR 0.28, 95% CI 0.13–0.63).

### Patterns of gene variants at baseline and effects on ORR

**Fig. 5:**
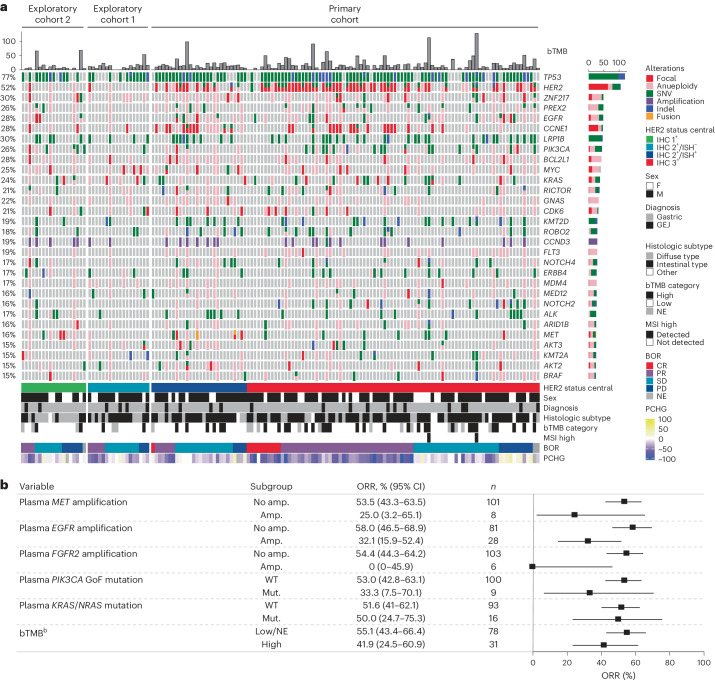
Genomic alterations as determined by ctDNA. **a**, Landscape of genomic alterations in baseline ctDNA^a^. **b**, Relationships among ctDNA-detected alterations in key signal transduction genes (*MET*, *EGFR*, *FGFR2*, *PIK3CA* GoF*, KRAS*/*NRAS*), tumor mutation burden and ORR. ^a^Excluding germline, putative clonal hematopoiesis of indeterminate potential and synonymous variants without annotations using OncoKB. Included any amplification type (focal and aneuploidy). Excluding two patients in exploratory cohorts because of missing HER2 test result. Highly frequent genes (≥15%) are shown. Patients were ordered by cohort, HER2 status, BOR and OS. ^b^bTMB cutoff value was 20 mutations per megabase according to the Guardant Health OMNI platform. **b**, Error bars represent 95% CI. BOR, best overall response; MSI, microsatellite instability; PCHG, percentage change from baseline (tumor size by computed tomography scan).

In pretreatment analysis of genomic alterations, as determined by ctDNA analysis, there was a high prevalence of alterations in *TP53* (77%, single nucleotide variants (SNVs) or insertions/deletions (Indels)), *HER2* (52%, 51% amplification (amp.)), *CCNE1* (28% amp.) and *EGFR* (28%, 22% amp., 10% SNVs/Indels) (Fig. 5a). Patients with plasma amplifications, defined according to the Guardant Health OMNI platform, in genes *MET*, *EGFR* and *FGFR2* had numerically lower ORR (Fig. 5b). ORR was 25% (95% CI 3.2–65.1%) in patients with *MET* amplifications (*n* = 8), 32.1% (95% CI 15.9–52.4%) in patients with *EGFR* amplifications (*n* = 28) and 0% (95% CI 0–45.9%) in patients with *FGFR2* amplifications (*n* = 6). ORR was 55.1% (95% CI 43.4–66.4%) in patients with low/NE blood tumor mutation burden (bTMB) (20 mutations per megabase cutoff value, according to the Guardant Health OMNI platform) (*n* = 78) and 41.9% (95% CI 24.5–60.9%) in patients with high bTMB (*n* = 31) (Fig. 5b). ORR was 50.0% (95% CI 24.7–75.3%) in patients with *KRAS*/*NRAS*-activating variants (*n* = 16) compared with 51.6% (95% CI 41.0–62.1%) in patients with wild-type (WT) *KRAS*/*NRAS* (*n* = 93) (Fig. 5b). A total of nine patients had *PIK3CA* GoF mutations, with an ORR of 33.3% (95% CI 7.5–70.1%), compared with 100 patients with WT *PIK3CA*, with an ORR of 53.0% (95% CI 42.8–63.1%) (Fig. 5b).

### Patterns of gene variants at EoT

**Fig. 6:**
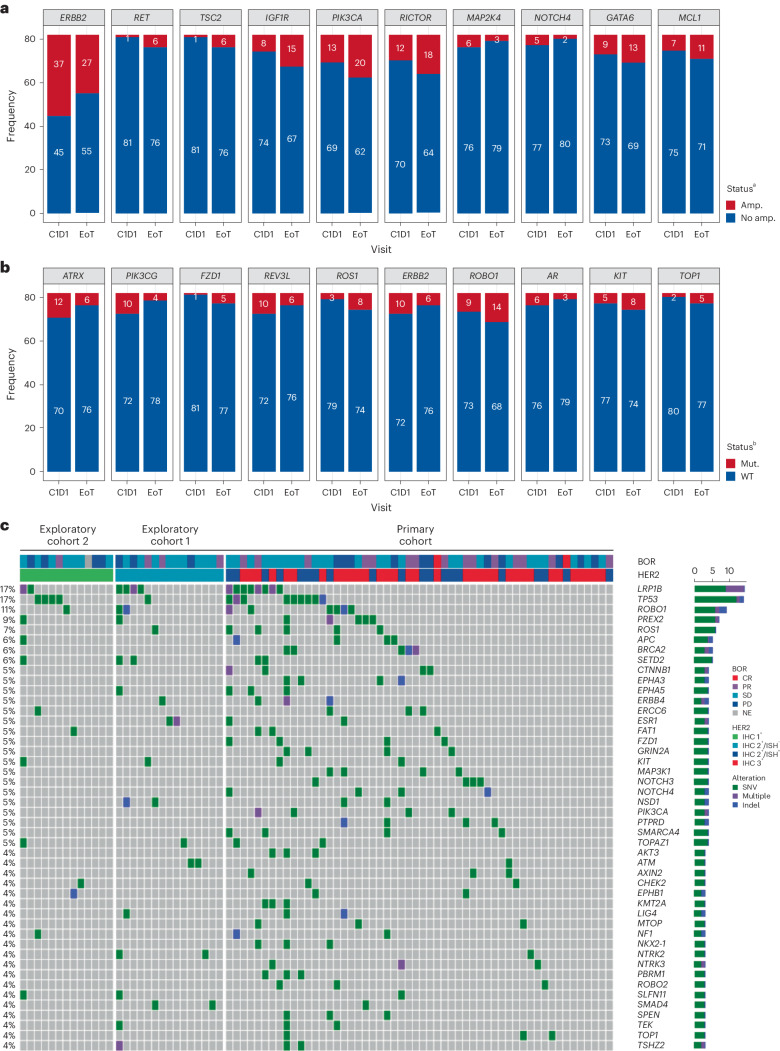
Analysis of patterns of gene variants at EoT. **a**,**b**, Acquired/lost gene alteration from baseline to EoT by comparison of the change in gene amplification status (**a**) and by comparison of the change of SNV/Indel status (**b**). **c**, SNVs/Indels and amplifications from EoT samples. ^a^Top ten acquired/loss of amplification from baseline to EoT, including both arms, ordered by *P* value. The McNemar test was used to compare change in gene amplification status from baseline to EoT regardless of amplification type. Shown is high-frequency amplification at either baseline or EoT in at least five patients among whom *HER2* was the only significant amplification change (*P* = 0.0064). Multiple testing correction was not performed. ^b^Top ten acquired/loss of SNVs/Indels from baseline to EoT, including both arms, ordered by *P* value. The McNemar test was used to compare change in SNV/Indel status from baseline to EoT regardless of function. Shown are high-frequency SNVs/Indels at either baseline or EoT in at least five patients. There were no significant SNVs/Indels. Multiple testing correction was not performed. ^c^The order of data is based on an algorithm aimed at determination of mutual exclusivity. The heatmap shows potential acquired SNVs/Indels detected only at EoT, regardless of function. C1D1, cycle 1, day 1.

**Extended Data Fig. 2:**
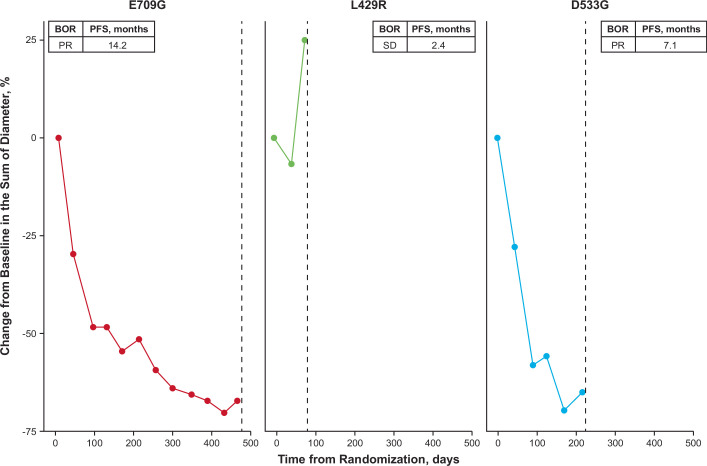
*TOP1* acquired mutations identified in 3 patients at end of treatment. BOR, best overall response; mo, months; PFS, progression-free survival; PR, partial response; SD, stable disease; TOP1, topoisomerase 1.

Although there were some changes in ctDNA SNVs/Indels and amplifications observed by the end of treatment (EoT), no common trends were observed (Fig. 6a,b). Before receiving T-DXd treatment, 45% (37 of 82) of patients with *HER2* gene expression had ctDNA amplification. However, this proportion decreased to 33% (27 of 82) at EoT. Similar variances were observed across other genes. Some EoT samples showed the presence of acquired *TOP1* variants (Fig. 6b and Extended Data Fig. 2) and acquired SNVs/Indels (Fig. 6c).

### Analysis of gene expression in responders and nonresponders

**Extended Data Fig. 3:**
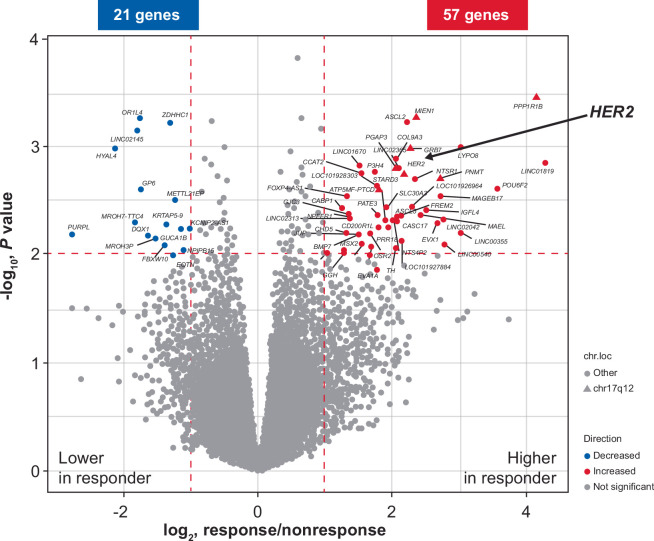
Differential expression of various genes in responders and nonresponders. chr.loc, chromosome location; HER2, human epidermal growth factor receptor 2; RECIST, Response Evaluation Criteria in Solid Tumors. Results are ordered by statistical value, and genes in Chr 17q12 region are shown as triangles. To identify differentially expressed genes between responder (complete response or partial response) and nonresponder (progressive disease, stable disease, not evaluable) defined by RECIST, moderated t-test was used without multiple testing correction (P ≤ 0.01, absolute log2 FC ≥ 1).

Several genes were found to be differentially expressed between responders and nonresponders following T-DXd treatment in the primary cohort, as defined by Response Evaluation Criteria in Solid Tumors (RECIST) (responder: complete response (CR) or partial response (PR); nonresponder: progressive disease (PD), stable disease (SD) or NE) (Extended Data Fig. 3). Overall, 57 genes showed higher expression in responders (absolute log_2_ fold change (FC) ≥ 1, *P* ≤ 0.01), including neighboring genes to *HER2* in the *HER2* amplicon (nine additional genes). Responders had significantly higher expression of genes on chromosome 17q12-21, the chromosome locus in which *HER2* is located. These genes of interest are likely to be coamplified with *HER2*, and higher expression of genes located in chromosome 17q12-21 was associated with better response. There was no obvious relationship between PD-L1 RNA level and T-DXd efficacy (log_2_ FC −0.41, *P* = 0.32).

## Discussion

These exploratory biomarker analyses of patients from the primary and exploratory cohorts of the DESTINY-Gastric01 trial identified relevant prognostic or predictive biomarkers in patients with HER2^+^ or HER2-low gastric and GEJ cancer treated with T-DXd. Patients with higher levels of HER2-associated biomarkers, including HER2 IHC positivity, HER2 ISH positivity, tumor *HER2* mRNA levels, plasma gene apCN and/or serum HER2ECD, had numerically higher ORR compared with patients with lower HER2-associated biomarkers. For example, patients with plasma *HER2* amplification in ctDNA had an ORR of 61% compared with 34% in patients with no amplification. These results suggest that T-DXd has activity even in patients with HER2-low tumors, which confirms previous findings from the HER2-low DESTINY-Gastric01 exploratory cohorts in which the confirmed ORR was 26.3% (95% CI 9.1–51.2%) and 9.5% (95% CI 1.2–30.4%) in the IHC 2^+^/ISH^−^ and IHC 1^+^ cohort, respectively^5^. However, some discordance was observed between plasma *HER2* amplification and HER2 IHC positivity in tumor tissue collected before T-DXd treatment in the current study (Extended Data Table 2). Although probably due to a technological limitation in detecting plasma *HER2* amplification or tumor heterogeneity, it is important to note there is a possibility that location of metastasis is related to levels of ctDNA^14^. This may also play a role in discordance between tumor and plasma biopsy results. However, NPA was high, which may support the use of ctDNA to guide treatment if there are no available tissue samples. Further investigation is warranted to validate whether plasma *HER2* amplification could replace tissue IHC/ISH in HER2 scoring.

In the current study, tumor *HER2* mRNA level, plasma gene apCN and HER2ECD level were all correlated with HER2 status in tumor biopsy samples assessed with IHC/ISH. Although *HER2* amplification and associated overexpression in gastric and GEJ cancer is poorly understood, our results might provide some insights into this relationship^15^. Alterations in other genes were found to overlap with *HER2* amplification in our analysis, as determined by ctDNA analysis at baseline. Although bTMB did not appear to have a major effect on response to treatment, patients with alterations in key signal transduction genes (*MET*, *EGFR*, *FGFR2* or *PIK3* GoF) had numerically lower ORR. There were trends for worse outcomes in patients with *MET*, *EGFR* and *FGFR2* amplification; however, interpretation is limited by the small number of patients with these alterations and the lack of a control arm in our study. It remains unknown whether these trends are related to resistance to T-DXd or are simply prognostic effects.

We found no significant relationship between PD-L1 RNA expression and T-DXd efficacy. However, given recent findings from the KEYNOTE-811 trial^16^, further investigations are warranted to explore the relationship between PD-L1 IHC, HER2 IHC and the efficacy of T-DXd. Our data suggested there might be higher ORR in the HER2-low gastric cancer cohort of patients with high levels of plasma HER2ECD compared with those with low HER2ECD levels. However, these results are not conclusive because of the small number of patients in the exploratory cohorts, and confirmation in additional HER2-low cohorts would be required for validation. This might be provided in the ongoing DESTINY-Gastric03 (NCT04379596) and EPOC2203 (jRCT2031230477) studies, which evaluated T-DXd combinations for HER2-low gastric cancer.

*HER2* ctDNA amplification was detected in approximately half of the patients in this study (45% (37 of 82)) before T-DXd treatment and in 33% of patients (27 of 82) by EoT. This is consistent with previous observations for other HER2-targeting therapies for gastric cancer such as trastuzumab and lapatinib^17,18^.

It is unlikely that HER2 expression is lost on a per-cell basis. Instead, it is possible that the tumor was heterogeneous at baseline, with the ratio of HER2-expressing to -nonexpressing cells changing during treatment, which could indicate that non-*HER2*-amplified cells predominated in the tumor. For these patients, switching to non-HER2-targeted therapy may be an appropriate choice; however, further investigation in this subgroup of patients would be required to confirm this.

A previous trial of T-DXd identified several variants in nonsmall cell lung cancer, including exon 20 insertions and variants at position 310 (ref. ^19^). In the current study, although we did not observe exon 20 insertions, there were variants at position 310. In addition, 11.0% (12 of 109) of patients had *HER2* GoF variants and all patients with *HER2* GoF variants had HER2 IHC 3^+^ or IHC 2^+^/ISH^+^ status. Patients with HER2 IHC 3^+^ and *HER2* mutation had a higher ORR (87.5%, seven of eight) than those with HER2 IHC 3^+^ in the overall population (58.2%, 53 of 91), suggesting that patients with *HER2* mutation may be more sensitive to T-DXd, even in those with HER2 IHC 3^+^ status. Analysis of ctDNA carried out at EoT identified three cases of acquired variants in the *TOP1* gene (E709G, L429R and D533G) (Extended Data Fig. 2). The *TOP1* gene encodes DNA topoisomerase I, which is the direct target of deruxtecan. Patients with triple-negative metastatic breast cancer reported variants in *TOP1* with resistance to sacituzumab govitecan^20^. Sacituzumab govitecan is an ADC comprising an anti-Trop2 conjugated to another DNA TOP 1 inhibitor^20^. Of the three variants identified, D533G was previously found to be resistant to DNA topoisomerase I inhibition by camptothecin^21^. Structural analysis indicated that the D533 residue is in direct contact with camptothecin when it forms a complex with DNA and topoisomerase 1 (ref. ^22^). The D533G mutation may also provide resistance to deruxtecan, which is a derivative of camptothecin, although this theory needs to be confirmed by in vitro experiments. The E709G and L429R mutants have not previously been reported and neither the effect of these two variants on DNA topoisomerase I enzymatic activity, nor on its inhibition by camptothecin or deruxtecan, is known^23^. Although the presence of these acquired *TOP1* mutations suggests that direct mutations of *TOP1* may contribute to T-DXd resistance through provision of deruxtecan resistance, this appears to be a relatively rare event. In patients with acquired deruxtecan resistance but with maintained HER2 positivity, additional therapy with a HER2-targeting ADC incorporating a different payload might overcome such resistance.

Our study identified several genes with differential expression in T-DXd responders compared with nonresponders. *HER2* and several other genes in the chromosome 17q12-21 region were expressed at higher levels in T-DXd responders compared with nonresponders. The expression level of those genes was associated with *HER2* apCN, and increased expression levels of the genes on chromosome 17q12-21 is probably due to overall amplification of this region. Ultimately, the association of these genes with patient outcomes may be a secondary effect of the genomic amplification of this region and *HER2* overexpression.

Results from studies in other cancer types have suggested that identification of specific expression patterns may provide prognostic benefit and allow for tailored therapeutic regimens^24^. The relationship between the timing of tumor sample collection used for assessment of HER2 IHC/ISH status and patient outcomes revealed that clinically meaningful ORR was observed regardless of the timing of sample collection in our study (that is, before or after/during the first Tmab treatment). HER2-targeted treatment can reduce the number of HER2^+^ cells (decreased HER2 expression following treatment with trastuzumab or other HER2-targeted agents has been observed in 16–32% of patients)^10–12,25^ and, consequently, confirmation of HER2^+^ following trastuzumab therapy is important before initiating another HER2-directed therapy. However, because the current analysis was from a third- or later-line study, there was a period of non-HER2-targeting therapy between the last dose of Tmab and initiation of T-DXd treatment. It is possible that HER2^+^ cells could regrow during this period of non-HER2-targeting therapy^11^. As the OS results suggest, HER2 status at baseline might be predictive of the efficacy of T-DXd. Therefore, although a fresh biopsy sample is useful for second-line therapy, it may have lower utility for third- or later-line therapy.

Limitations of these biomarker analyses include the fact that they were either exploratory or post hoc analyses with a small sample size conducted in the absence of external validation and data from a control arm. However, one strength of these exploratory analyses is that the data are from a robustly designed clinical trial of T-DXd compared with chemotherapy in patients with HER2^+^ advanced gastric cancer; the analyses presented are from patients in the T-DXd arm of the primary cohort as well as from those in the two exploratory cohorts, all of whom received T-DXd^4^. The biomarkers identified in this analysis are being investigated and validated in additional studies, including DESTINY-Gastric02 (ref. ^6^), DESTINY-Gastric03 (NCT04379596), DESTINY-Gastric04 (NCT04704934) and EPOC2203 (jRCT2031230477).

## Methods

### Study design and patient demographics

DESTINY-Gastric01 (NCT03329690) was an open-label, multicenter, randomized, phase 2 trial that included patients with HER2-expressing advanced gastric or GEJ carcinoma that had progressed on two or more previous lines of therapy, including fluoropyrimidine, a platinum agent and trastuzumab^4^. Patients enrolled in the primary cohort had HER2^+^ tumors, as determined by IHC or ISH positivity (IHC 3^+^ or IHC 2^+^/ISH^+^). Patients received either T-DXd at 6.4 mg kg^−1^ every 3 weeks (*n* = 125) or TPC (irinotecan or paclitaxel; *n* = 62). Patients enrolled in the exploratory cohorts had HER2-low expression and were anti-HER2 treatment naive^5^. Patients in exploratory cohort 1 were HER2 IHC 2^+^/ISH^−^ (*n* = 20) and those in exploratory cohort 2 were HER2 IHC 1^+^ (*n* = 24), and all received T-DXd 6.4 mg kg^−1^ every 3 weeks. The primary endpoint was ORR by independent central review, with secondary endpoints OS, duration of response, progression-free survival, confirmed ORR (ORR lasting ≥4 weeks) and safety. Responders and nonresponders were defined by RECIST (responder: complete response or partial response; nonresponder: progressive disease, stable disease or NE).

The study was designed and supervised by Daiichi Sankyo. Written informed consent was provided by all patients before enrollment. An independent ethics committee or institutional review board at each site reviewed and approved the protocol (online only). The study was conducted according to the study protocol, in accordance with the principles of the Declaration of Helsinki, the International Conference on Harmonization Guidelines for Good Clinical Practice and other local regulations where applicable.

### Sample collection and biomarker assay

Exploratory biomarker data were collected from patients in the T-DXd arm of the primary cohort and from the exploratory cohorts (Fig. 1). At baseline, RNA-seq was used to examine biomarkers from tumor biopsies for *HER2* gene expression, a comprehensive gene expression profile and an immune-related gene profile. IHC (4B5, Ventana Medical Systems, Inc.) and ISH were used to confirm *HER2* expression and genome status. Total RNA extraction was conducted using the Qiagen RNeasy Mini Kit at Labcorp. RNA-seq libraries were prepared using the NEBNext Ultra II Directional RNA library Prep Kit for Illumina and NEBNext Multiplex Oligos for Illumina according to laboratory methods, and sequencing was performed with Illumina NovaSeq 6000 or NextSeq including 75 base pairs from each end (2 × 75 base pairs) performed at Daiichi Sankyo. Sequencing reads were aligned with STAR software (2.5.3a) to human genome reference GRCh38. The number of transcripts per million and the expected count in each gene were estimated by RNA-seq using expectation-maximization software (1.3.0). Gene expression levels are shown as log_2_ counts per million. For RNA-seq data, the expression value of each gene was normalized using the voom method, and a linear model was used to detect differentially expressed genes between responders and nonresponders. Liquid biopsy was used for ctDNA assay of plasma with the Guardant OMNI panel (Guardant Health) to examine plasma *HER2* amplification, *HER2* copy number, gene alterations and bTMB as provided in the Guardant Health OMNI platform. Samples with no somatic mutations detected in any gene in the Guardant OMNI assay were excluded from ctDNA analysis. All possible germlines and clonal hematopoiesis of indeterminate potential reported by Guardant Health OMNI platform were excluded. SNVs/Indels were annotated using OncoKB, and unannotated variants with low variant allele frequency (≤0.2) were excluded in this analysis^26^.

*HER2* plasma copy number was adjusted by maximum variant allele fraction as a tumor fraction in each individual sample according to a previous publication^27^. HER2ECD (ng ml^−1^) was measured in serum samples by ADVIA Centaur HER2/neu assay (Siemens Medical Solutions Diagnostics), a *HER2*-associated liquid biomarker. ctDNA analysis was also conducted to compare genetic alterations in patients (*n* = 82) who had discontinued treatment because of progressive disease or clinical progression between baseline and EoT. The McNemar test determined the possible acquired mutations involved with the resistance of T-DXd among these patients.

### Statistical analyses

Point estimates and two-sided 95% exact binomial CIs were calculated for ORR in each subgroup. The Kaplan–Meier method was used to estimate median event times, with two-sided 95% CIs calculated using Brookmeyer and Crowley methods. HRs with two-sided 95% CIs were estimated using Cox proportional-hazards regression models. Exploratory cutoff values (plasma copy number of 6.0, apCN of 18.2 and HER2ECD of 14.4 in the primary cohort; *HER2* mRNA gene expression of 7.7 and HER2ECD of 11.6 in the exploratory cohorts) were selected based on the most significant value (OS) for separation of patients into high and low groups. The McNemar test was used to compare ctDNA mutational status changes between baseline and EoT.

### Reporting summary

Further information on research design is available in the Nature Portfolio Reporting Summary linked to this article.

## Online content

Any methods, additional references, Nature Portfolio reporting summaries, source data, extended data, supplementary information, acknowledgements, peer review information; details of author contributions and competing interests; and statements of data and code availability are available at 10.1038/s41591-024-02992-x.

### Supplementary information

Supplementary Information Supplementary Table 1 with additional data-sharing information.

Reporting Summary

## Figures and Tables

**Extended Data Table 1:**
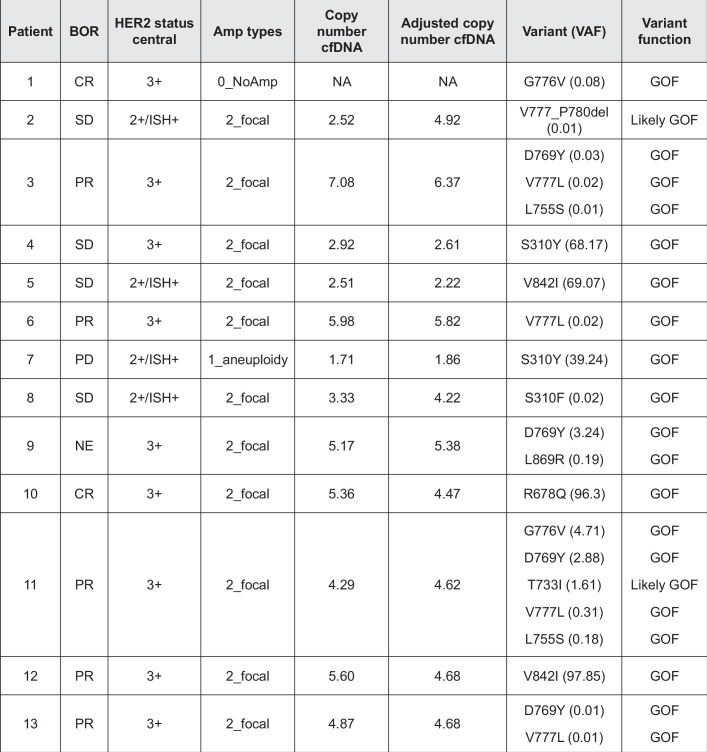
HER2 GOF variants in individual patients

Amp, amplification; BOR, best overall response; cfDNA, circulating free DNA; CR, complete response; HER2, human epidermal growth factor receptor 2; GOF, gain-of-function; ISH, in situ hybridization; NE, not estimable; PR, partial response; SD, stable disease; T-DXd, trastuzumab deruxtecan; VAF, variant allele frequency. Variant types overlap with HER2 amplification. All patients are from the primary cohort T-DXd arm.

**Extended Data Table 2:**
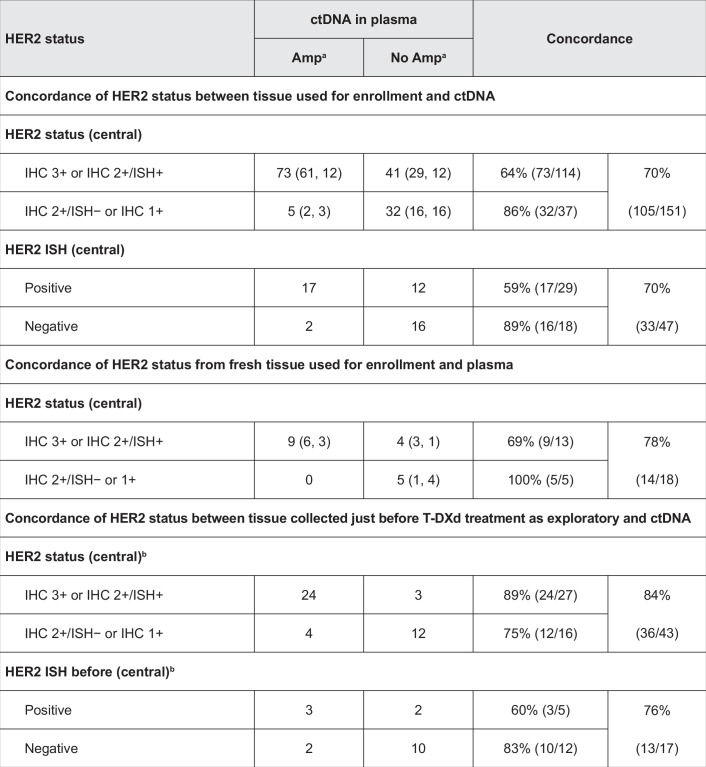
Concordance of HER2 status between tissue and plasma

Amp, amplification; ctDNA, circulating tumor DNA; HER2, human epidermal growth factor receptor 2; IHC, immunohistochemistry; ISH, in situ hybridization; T-DXd, trastuzumab deruxtecan.

^a^Includes both types of Amp, focal and aneuploidy; data are shown as number of patients regardless of amplification type (focal, aneuploidy).

^b^All samples were from archival tissue and centrally tested for HER2 status before T DXd treatment.

## Data Availability

Please see Supplementary Table 1 for further details on data availability. Anonymized individual participant data on completed studies and applicable supporting clinical study documents may be made available upon request at https://vivli.org/. In cases where clinical study data and supporting documents are provided pursuant to our company policies and procedures, Daiichi Sankyo Companies will continue to protect the privacy of the company and our clinical study patients. Details on data-sharing criteria and the procedure for requesting access can be found at this address: https://vivli.org/ourmember/daiichi-sankyo/.
