# Supplementary material for: Trastuzumab deruxtecan in HER2-positive advanced gastric cancer: exploratory biomarker analysis of the randomized, phase 2 DESTINY-Gastric01 trial
Source: Nat Med. 2024 May 14;30(7):1933–42. doi: 10.1038/s41591-024-02992-x (PMC11271396; doi:10.1038/s41591-024-02992-x)
Supplement: Supplementary file 1 — Supplementary Table 1 with additional data-sharing information. [file 41591_2024_2992_MOESM1_ESM.pdf]

nature medicine

Article https://doi.org/10.1038/s41591-024-02992-x

Trastuzumab deruxtecan in HER2-positive advanced gastric cancer: exploratory biomarker analysis of the randomized, phase 2 DESTINY-Gastric01 trial

In the format provided by the authors and unedited

Additional data sharing information

Data sharing questions	Answers
Will individual participant data be available (including data dictionaries)?	Yes
What data in particular will be made available?	Anonymised individual participant data (IPD) on completed studies and applicable supporting clinical trial documents may be available upon request at https://vivli.org/. In cases where clinical trial data and supporting documents are provided pursuant to our company policies and procedures, Daiichi Sankyo, Inc., will continue to protect the privacy of our clinical trial participants. Details on data sharing criteria and the procedure for requesting access can be found at this web address: https://vivli.org/ourmember/daiichi-sankyo/.
What other documents will be available?	Clinical Trial Protocol, Statistical Analysis Plan, Informed Consent Form, and Clinical Study Report. In cases where clinical trial data and supporting documents are provided pursuant to our company policies and procedures, Daiichi Sankyo will continue to protect the privacy of our clinical trial participants.
When will data be available (start and end dates)?	Anonymised IPD will be available when indication supported receives marketing approvals and study results are published.
To whom with data be available?	Qualified science and medical researchers upon formal request and submission of research proposal detailing planned analyses.
For what type of analyses?	De-identified IPD and relevant clinical trial documents will be shared for the purpose of conducting legitimate research as specified in an approved formal research proposal.
By what mechanism will data be made available?	De-identified IPD will be available upon request at https://vivli.org/.
